# Feasibility and preliminary efficacy of the LEAD trial: a cluster randomized controlled lifestyle intervention to improve hippocampal volume in older adults at-risk for dementia

**DOI:** 10.1186/s40814-022-00977-6

**Published:** 2022-02-09

**Authors:** N. D. Koblinsky, N. D. Anderson, F. Ajwani, M. D. Parrott, D. Dawson, S. Marzolini, P. Oh, B. MacIntosh, L. Middleton, G. Ferland, C. E. Greenwood

**Affiliations:** 1 Rotman Research Institute, Baycrest Health Sciences, Toronto, Canada; 2 Departments of Psychology and Psychiatry, University of Toronto, Toronto, Canada; 3 KITE, Toronto Rehabilitation Institute – the University Health Network, Toronto, Canada; 4 PERFORM Centre, Concordia University, Montreal, Canada; 5 Department of Occupational Sciences and Occupational Therapy and Rehabilitation Sciences Institute, University of Toronto, Toronto, Canada; 6 Hurvitz Brain Sciences, Sunnybrook Research Institute, University of Toronto, Toronto, Canada; 7 University of Waterloo, Waterloo, Canada; 8 Montreal Heart Institute Research Centre, Montreal, Canada; 9 Department of Nutrition, Université de Montréal, Montreal, Canada; 10 Department of Nutritional Sciences, University of Toronto, Toronto, Canada

**Keywords:** Exercise, Diet, Intervention, Feasibility, Subjective cognitive decline, Early MCI, Vascular risk factors

## Abstract

**Background:**

Healthy diet and exercise are associated with reduced risk of dementia in older adults. The impact of diet and exercise interventions on brain health is less consistent, especially with dietary interventions which rely on varying approaches. Our objective was to evaluate the feasibility and preliminary efficacy of a 6-month intervention combining exercise with a novel dietary counseling approach to improve hippocampal volume among older adults at-risk for dementia.

**Methods:**

Participants with vascular risk factors and subjective cognitive decline or early mild cognitive impairment were cluster randomized in groups of 3–4 to the diet intervention (DIET) or control education (ED) group. All participants engaged in 1 h of supervised exercise per week and additional exercise at home. DIET involved 1 h per week of group-based dietary counseling comprising education, goal setting, and strategy training. ED involved 1 h per week of group-based brain health education classes. Our primary outcome was change in hippocampal volume from baseline to 6 months. Secondary outcomes included changes in cognitive function, blood biomarkers, diet, and fitness. Recruitment challenges and early discontinuation of the trial due to COVID-19 necessitated a revised focus on feasibility and preliminary efficacy.

**Results:**

Of 190 older adults contacted, 14 (7%) were eligible and enrolled, constituting 21% of our recruitment target. All participants completed the intervention and attended 90% of exercise and DIET/ED sessions on average. All 6-month assessments prior to COVID-19 were completed but disruptions to in-person testing resulted in incomplete data collection. No serious adverse events occurred and all participants expressed positive feedback about the study. Preliminary findings did not identify any significant changes in hippocampal volume; however, substantial improvements in diet and HbA1c were observed with DIET compared to ED (*d* = 1.75 and 1.07, respectively).

**Conclusions:**

High adherence and retention rates were observed among participants and preliminary findings illustrate improvements in diet quality and HbA1c. These results indicate that a larger trial is feasible if difficulties surrounding recruitment can be mitigated.

**Trial registration:**

ClinicalTrials.gov identifier: NCT03056508.

**Supplementary Information:**

The online version contains supplementary material available at 10.1186/s40814-022-00977-6.

## Key messages regarding feasibility

What uncertainties existed regarding feasibility?

- Limited evidence on recruitment, retention, and adherence for older adults with early dementia risk participating in combined exercise and diet interventions.
- The ability of a novel dietary intervention involving goal setting and strategy changing to promote adherence to a brain-healthy diet.

What are the findings on feasibility of this study?

- A small portion of people contacted met inclusion criteria, and the time commitment related to study visits deterred many people from participating.
- Enrolled participants demonstrated high retention, visit adherence, and satisfaction.
- The DIET intervention resulted in improved adherence to a brain-healthy diet.

What are the implications for the design of the main study?

- Broader inclusion criteria may be warranted to reach recruitment targets.
- Outcome assessments and visit requirements need to be reduced to lessen participant burden.
- DIET is a feasible intervention for a larger trial investigating the effects of a brain-healthy diet on brain structure and function.

## Background

Alzheimer’s disease and related dementias are among the world’s most prevalent and costly medical conditions [1]. An at-risk population for developing dementia are older adults with subjective cognitive decline (SCD) or early mild cognitive impairment (MCI). Individuals with SCD are concerned that their memory or thinking has declined despite no objective signs of cognitive impairment [2, 3]. SCD is reported by 50–80% of adults over 70 [4], with approximately 27% progressing to mild cognitive impairment and 14% progressing to dementia over 4 or more years [5–8]. Early MCI is defined by the Alzheimer’s Disease Neuroimaging Initiative (ADNI) as individuals who are less amnestic than those with late MCI and progress more slowly to dementia [9]. Approximately 50% of older adults do not meet physical activity requirements [10] and in a recent study, 95% of older adults with SCD reported difficulties in taking up exercise and healthy diet [11]. Vascular risk factors resulting from unhealthy lifestyle commonly contribute to cognitive decline [12]. In the absence of disease-modifying therapy, there is interest in mitigating cognitive decline through lifestyle modification. Older adults with SCD or early MCI and vascular risk factors in particular may benefit from lifestyle intervention before irreversible changes in brain health occur.

Exercise and adhering to a healthy diet are strongly associated with reduced dementia risk (30–60%) in cross sectional and longitudinal studies [13–18]. The intervention literature is less consistent, especially for dietary interventions which rely on varying approaches of dietary counseling [19]. Randomized controlled trials of exercise across a range of modalities, frequencies, and intervention durations suggest small to moderate effects on cognition among older adults [20]. The actions by which exercise affect the brain are suspected to be both indirect, such as improving health conditions, as well as more direct mechanisms, including increasing brain neurotrophic factors [21], improving cerebrovascular function [22], and enhancing brain plasticity [23]. Exercise programs that combine aerobic and resistance training may impact cognition over and above either alone [20].

Several high-quality trials have demonstrated a small effect of diet on cognition [24, 25], while other trials using various intervention modalities resulted in inconsistent adherence and findings [26–29]. Many trials relied on education alone and infrequent (bi-weekly to quarterly) counseling, which is likely insufficient to promote eating changes. There is a general consensus that it is the global diet attributes (i.e., high in fruits, vegetables, nuts, whole grains and fish, as well as reducing consumption of saturated fats, sodium, and highly processed foods) that are important for brain health rather than individual foods or nutrients [30]. Proposed mechanisms include reduced inflammation and oxidative stress [31] as well as neurogenesis and improved neuronal connectivity [32]. High adherence to a healthy diet is necessary for determining its effects on cognition [33]. Thus, it is important to understand how to optimize intervention delivery to achieve dietary change in at-risk populations.

Given the complex nature of dementia, interventions targeting several risk factors simultaneously may be necessary to achieve optimal effects [34]. We were particularly interested in whether a healthy diet intervention, when combined with exercise, would lead to better outcomes than an exercise intervention alone. The purpose of this study was to investigate the effects of a 6-month intervention combining exercise with a novel dietary counseling approach compared to exercise paired with a placebo education program on hippocampal volume in older adults with vascular risk factors and early dementia risk. Slow recruitment and early discontinuation of the trial due to the COVID-19 pandemic resulted in a revised focus on feasibility and preliminary efficacy. Feasibility measures included recruitment, retention, adherence, and safety. Effect sizes are reported to describe between group differences in outcome measure change from baseline to 6 months.

## Methods

### Study design

The Lifestyle, Exercise and Diet (LEAD) trial took place in Toronto, Canada, from July 2018 to July 2020. Participants were cluster randomized and equally allocated into a group-based diet intervention called the Baycrest Brain-healthy Eating Approach (DIET) or a placebo education program focusing on brain aging and tips to support brain health (ED). All participants engaged in 1 h of supervised exercise per week and additional exercise at home. DIET encompassed didactic nutrition education regarding a specific brain-healthy dietary pattern combined with goal setting and strategy training to promote sustainable dietary change [35]. ED acted as a time-matched placebo and was designed to be of equal frequency, duration, and social interaction/engagement as DIET. Outcomes were assessed at baseline, 6 months (post-intervention), and 12 months (follow-up).

The LEAD trial was a sub-study of the Canadian Consortium on Neurodegeneration in Aging (CCNA; ccna-ccnv.ca) and participants were enrolled in the CCNA Comprehensive Assessment of Neurodegeneration and Dementia (COMPASS-ND) study (NCT03402919). COMPASS-ND is a longitudinal study involving comprehensive clinical and neuropsychological testing, as well as neuroimaging to determine cohort membership (e.g., healthy control, SCD, MCI, and various forms of dementia) at baseline and at 2-year follow-up [36]. Refer to Additional file 2: Appendix 1 for a more detailed list of the COMPASS-ND procedures. Participation in the LEAD trial was contingent on first completing the COMPASS-ND baseline assessments which took place over 4 visits at either the Centre for Memory and Aging or Baycrest memory clinic in Toronto. The LEAD trial was a collaborative effort between Baycrest Hospital, Sunnybrook Hospital, and University Health Network’s Toronto Rehabilitation Institute, all situated in the large urban city of Toronto, Ontario, Canada. Baycrest was the lead site, responsible for recruitment. The intervention sessions took place at Toronto Rehabilitation Institute’s comprehensive Cardiovascular Prevention and Rehabilitation Program.

### Sample size

Sample size was originally estimated to compare the effect size associated with pre-post changes in hippocampal volume as a function of group. Based on an expected hippocampal volume effect size of 0.27, 3 time points, 2 groups, power = 0.9, alpha = 0.05, and correlation between repeated measures = 0.5, we required a minimum sample size of 60. The total sample size of 66 was used to accommodate a retention rate of approximately 90% and allow for the following covariates: inflammatory cytokines, oxidative burden, BDNF/APOE status and sex. Our effect size was derived from neuroimaging work that reported a hippocampal volume increase in a time by group analysis among older sedentary adults [37] and is comparable to other older adult exercise studies with functional [38], hemodynamic [39], and cognitive [40] outcome measures.

### Participants

#### Recruitment

Participants were recruited from the community through a volunteer database at Baycrest, investigator talks, community advertisements, participating memory clinics, and the Cardiovascular Prevention and Rehabilitation Program. Following telephone contact and screening, participants attended the initial COMPASS-ND assessments to confirm eligibility and provide written consent to participate in COMPASS-ND and LEAD.

#### Inclusion criteria

Participants were aged 60–85 years old with SCD or early MCI and ≥ 2 vascular risk factors. The initial COMPASS-ND visit was used to establish a research diagnosis. SCD was determined by answering “Yes” to the following Jessen questions: “Do you feel like your memory or thinking is becoming worse?” and “Does this worry you?” [2, 3]. They also had minimal or no cognitive deficit as indicated by having (1) a delayed recall score on Story A of the Logical Memory subtest of the Wechsler Memory Scale—Revised [41] above the ADNI education-adjusted cutoffs (= 9 for 16+ years of education; = 5 for 8–15 years of education; = 3 for 0–7 years of education); (2) a Montreal Cognitive Assessment (MoCA) total score above 24 [42]; (3) a delayed recall score on the Consortium to Establish a Registry for Alzheimer’s Disease (CERAD) word list above 5; and (4) a global Clinical Dementia Rating (CDR) score lower than 1.0. Although our original plan was to recruit only people with SCD, we expanded our inclusion criteria to include EMCI. The ADNI criteria [9] were used to determine EMCI, and included individuals who scored between 20 and 24 on the MOCA and 5 on the CERAD. Participants were required to possess ≥ 2 of the following vascular risk factors: overweight (BMI > 25), or physician diagnosis of the following conditions: type 2 diabetes mellitus or pre-diabetes (HbA1c ≥ 6.0% [43];); high cholesterol (total cholesterol > 240 mg/dL or LDL > 160 mg/dL); or hypertension (> 140/90 mmHg). They were also required to be reasonably inactive at baseline (less than 75 min per week of moderate or vigorous intensity physical activity assessed using the Godin Leisure Time Exercise Questionnaire [44]), be consuming a reasonably poor quality diet (a score of 2 or less on the Diet Screening Questionnaire, Additional file 2: Appendix 2), and be available for the whole intervention. Exclusion criteria were: significant known chronic brain disease (e.g., traumatic brain injury, epilepsy, Parkinson’s disease); major depression or clinical anxiety disorders; major psychiatric disorders; ongoing alcohol or drug abuse; inability to undergo an MRI scan; or contraindications to an exercise program [45].

### Randomization and blinding procedure

Our aim was to recruit groups of 4–6 older adults at a time from the community. Once a group was screened and had consented to participate, they were cluster randomized into either DIET or ED. Randomization was generated using the Random.org online software by the trial PI who was not involved in participant enrolment or assessment. The group allocation was then provided to the research coordinator who assigned participants to their groups. Cluster randomization was selected for feasibility reasons as the group-based format required 4–6 participants to begin the intervention and attend sessions concurrently. Screening visits for 4–6 participants (which included neuroimaging) were anticipated to take 2–3 weeks due to available CCNA staff and resources, which we considered to be an allowable time to wait before starting the intervention. It would not have been feasible to recruit and screen the 8–12 participants needed for simple or block randomization within this time frame. Assessors were blinded to group assignment and participants were asked not to mention their group assignment to assessors. To keep participants blinded from the hypotheses and group effect expectations, the content of the intervention and the wording of recruitment documents and consent forms did not convey the differences between DIET and ED conditions. Participants were specifically told that the groups would differ in the type of nutrition and brain health education that was provided.

### Intervention

#### Baycrest Brain-healthy Eating Approach (DIET)

DIET encompassed didactic nutrition education, goal setting, and strategy training to promote sustainable dietary change (Table 1). We first defined a dietary pattern which drew on scientific evidence linking diet to improved cognitive functioning in older adults with vascular risk factors. The recommended dietary pattern encompassed key features of two dietary interventions associated with cognitive benefits as implemented in the PREDIMED [24] and ENCORE [25] trials. Since neither trial published complete nutritional intake data for their cognitive subsamples, targeted and achieved levels of nutritional intake data from the larger trial populations were compared and formed the basis of the recommendations [46, 47]. In some cases, evidence from prospective cohort studies, published at the time, was used to further refine the recommendations. The principal foods or food groups that were encouraged were total vegetables; raw or leafy green vegetables; cruciferous vegetables; total fruit; berries; unsalted nuts or all natural butters with an emphasis on walnuts; fish or seafood; fatty fish; canned beans or cooked dried beans. Participants were recommended to limit their intake of meat and poultry; red or processed meat; butter, cream, or high fat dairy spreads; white bread; and processed foods. A translational product called the Brain Health Food Guide (Additional file 2: Appendix 3 [48]) was created to educate individuals on the diet recommendations.

**Table 1:** Key elements in the Baycrest Brain-healthy Eating Approach

Element	Description
1. Brain Health Food Guide	The guide was based on existing European and American epidemiologic and clinical trial results related to diet and cognitive function or dementia risk. It was designed as a practical, lifelong eating guide that does not eliminate any type of food but emphasizes variety and moderation, and offers maximum flexibility for individual food choices and preferences. Recommendations on daily or weekly servings of foods to include and foods to limit is provided as part of the guide.
2. Didactic Education	Participants selected topics of interest and were provided details about the health benefits of particular foods, suggestions for how these foods might be incorporated into one’s diet, and making healthy food choices. Topics selected were incorporating legumes into one’s diet, the health benefits of various plant foods, healthy fats, and label reading
3. Individual Nutritional Counseling	Each participant was offered a 30-min session with the dietitian to address individual concerns and barriers to change. Participants missed 30-min of group discussion to receive their individual counseling. If participants felt they needed to schedule additional individual sessions, they were accommodated on a case by case basis.
4. Individualized Goal Setting	Each week, participants selected a dietary change to align their diets more closely to the Brain Health Food Guide and put it into a goal statement.
5. Meta-cognitive strategy us	The meta-cognitive strategy, GOAL-PLAN-DO-CHECK, provides the frame for behaviour change.
6. Guided Discovery	This method of instruction is accomplished through having the group leader act as a facilitator rather than instructing participants. The group leader uses a series of questions to help participants identify plans that will achieve their goals in a way that will work for them and encourages participants to focus on “one thing at a time”.
7. Dynamic Performance Analysis	While facilitating the plan development, the group leader encourages the active analysis of whether the proposed plan is feasible and do-able. Thus, the planning is an iterative process.
8. Intervention Format	1-hour facilitated session that included goal setting, education, a brain-healthy snack break and group support.

The DIET intervention was built upon a previously established Cognitive Orientation to Daily Occupational Performance (COOP) Approach which was designed to promote skill acquisition and facilitate activity engagement in older adults [49]. COOP has previously shown to be a valid strategy to assist with lifestyle changes in older adults [50]. For our study, COOP was renamed Adult Strategies Put Into Real World Environments (ASPIRE) to describe the approach when applied to specific lifestyle goals. The COOP elements of guided discovery, goal setting, and meta-cognitive strategy were adapted in ASPIRE to specifically support the goals of the DIET intervention. Once per week, participants attended a 1-h group session with the study dietitian (Additional file 2: Appendix 4) who underwent formal training by individuals with expertise in this approach. The dietitian used guided discovery to help participants develop and adapt their own goals and identify ways to overcome barriers to implementation. Participants took part in an iterative process of working to attain their personalized diet goals using a meta-cognitive strategy (Goal-Plan-Do-Check). Participants self-selected goals on a weekly basis, which were rationalized within the context of the Brain Health Food Guide and individuals were expected to incrementally improve an aspect of their diet on a weekly basis. The didactic education curriculum included some set topics around the Brain Health Food Guide, but also many open sessions where participants were given the chance to pick topics that would help them move toward their goals. There were also brainstorming sessions where the group came up with ideas, and the dietitian only acted as a facilitator, not education provider. Results from the baseline Canadian Diet History Questionnaire II (C-DHQ II [51];), describing dietary intake patterns, were distributed to participants during the first or second session to help participants identify areas where their diet could be improved. Twice throughout the second half of the program, participants were allowed to schedule individual meetings with the study dietitian. If participants felt they needed to schedule additional individual sessions, they were accommodated on a case by case basis. Participants submitted weekly logs outlining diet goals, plans, successes, and obstacles to help directly identify sequential goals. Every month, participants were asked to complete an eating pattern self-assessment to help them assess the degree of dietary change achieved (Additional file 2: Appendix 5).

#### Brain health education sessions (ED)

Once per week, participants in the ED group participated in a 1-h group discussion and education session about lifestyle practices to support brain health (Additional file 2: Appendix 4). The ED program was adapted from the placebo program used by our partner CCNA sub-study ENGAGE (NCT#03271190 [52];). These classes were designed to be of equal frequency, duration, and social interaction/engagement compared to DIET sessions and were manualized to ensure consistency between trainers. During the classes, participants received information on brain and cognitive processes, the effect of age on cognition, and tips to promote successful aging. Twice throughout the second half of program, participants were allowed to schedule individual meetings with a study dietitian. Individuals in these groups did not participate in strategy training to assist them in undertaking dietary changes or receive the supplemental Brain Health Food Guide information.

#### Exercise

Exercise took place at Toronto Rehabilitation Institute’s Cardiovascular Rehabilitation and Prevention Program in Toronto, Ontario. This clinical program has demonstrated success in multiple vascular cohorts, notably coronary artery disease [53, 54]. The program is led by a team of physicians, physiotherapists, nurses, kinesiologists, psychologists, and dietitians. Study participants were enrolled in the program which included aerobic and resistance training, exercise education/counseling, and goal setting. Participants were required to complete 5 aerobic and 2–3 resistance training sessions per week. Participants attended 60-min group supervised exercise sessions once per week, with the remainder of the exercise sessions being completed in the home/community. Thirty minutes of exercise education was provided before every supervised exercise session. Embedded into the exercise education sessions were 2.5 h total of formal nutrition education which addressed fats, fiber, sodium, and food labels. Study participants also had access to clinical program’s psychosocial counseling upon request.

The initial aerobic exercise prescription was based on the results of a baseline cardiopulmonary assessment and was re-evaluated throughout the program. The initial intensity was equivalent to the anaerobic threshold and/or 60–80% of V̇O_2peak_ (mL kg^−1^ min^−1^) and was increased to a maximum of 80% of V̇O_2peak_. Resistance training was started in week 8 of the program, and was performed 2 to 3 times per week (1–2 times at home in an unsupervised setting). Resistance was provided by hand-held dumbbells, the patient’s body weight, and elastic bands of different thickness. Exercises included 3 lower body, 5 upper body, and 2 trunk-stabilizing exercises.

Participants were required to log each exercise session, noting the distance walked/jogged, duration, resting and peak heart rate, Borg rating of perceived exertion [55], and any symptoms experienced during exercise. Participants were trained to measure resting and exercise heart rates. Resistance training logs included the amount of weight lifted, and the number of repetitions and sets performed for each workout. This record was submitted and cross validated by an exercise specialist during the participant’s weekly visit.

### Outcome measures

#### Feasibility

Feasibility measures included recruitment, retention, adherence, and safety. We report data on recruitment sources and the number of participants screened and enrolled. Retention rate is reported as the proportion of participants who completed the intervention and the proportion of participants who completed 6- and 12-month assessments. Retention through follow-up is reported to show how many participants completed the assessments before COVID-19 versus during COVID-19. Adherence was measured as the percentage of intervention sessions attended. We also report the percentage of exercise logs and diet self-assessments completed and the number of one-on-one sessions with the dietitian that were scheduled. Following the intervention, participants were asked to complete an anonymous feedback questionnaire (Additional file 2: Appendix 6,7,8) about knowledge/skills gained, the length of sessions, what they found most or least useful, and whether they would recommend the study to other individuals concerned about their memory.

#### Preliminary efficacy

LEAD outcome measures included structural and functional MRI, cognitive function, fasting blood biomarkers, anthropometric measurements, dietary adherence, physical fitness, gait and balance, and a variety lifestyle and psychosocial questionnaires (Additional file 2: Appendix 9). Assessments were spread over 3 visits of approximately 2–3 h each. These visits took place at Sunnybrook hospital except for fitness testing and blood draws which were done at the Cardiovascular Prevention and Rehabilitation Program and the memory clinics, respectively. During COVID-19, participants were asked to participate in a shortened assessment which included an MRI, remote cognitive assessment, blood draw, graded exercise test, and online diet assessment. These measures were the focus of our preliminary efficacy analyses.

#### MRI

MRI data were collected according to the Canadian Dementia Imaging Protocol [56]. A 60-min brain MRI protocol consisted of (1) high spatial resolution anatomical imaging sequences; (2) an attention-related task-based blood oxygenation level dependent (BOLD) functional MRI (fMRI) that involved button responses to visual stimuli during a Flanker test; (3) two functional sequences during a resting state, one of which used BOLD contrast while the other used arterial spin labeling for cerebral blood flow; and (4) additional structural sequences to assess small vessel disease and white matter integrity, i.e., fluid attenuated inversion recovery and diffusion tensor imaging, respectively. The sequences are as follows: 3D T1-weighted MRI, PD/T2-weighted MRI, FLAIR, Gradient Echo, Resting State fMRI (BOLD), DTI, PCASL resting, Attention-based task fMRI (BOLD).

Images were acquired on a 3 Tesla Siemens Prisma scanner with a 12-channel head coil. Each participant’s head was restrained using cushions that fit inside the head coil. High-resolution structural images (T1-weighted three-dimensional magnetization-prepared rapid gradient-echo sequence; 3D-MPRAGE) were acquired with the following parameters: TR/TE = 2300/2.98, FOV = 256 mm, slice thickness = 1 mm, number of slices = 192. Whole hippocampal segmentation was performed using an established deep learning HippMapp3r algorithm (hippmapp3r.readthedocs.io) that was based on a convolutional neural network [57]. It uses a T1-weighted image as the only input and the outputs are segmentation masks for the left and right hippocampus.

#### Cognition

Our cognitive testing session was 2–3 h and was harmonized with the COMPASS-ND neuropsychological battery and a concurrent CCNA sub-study (ENGAGE: NCT#03271190 [52];). In addition to the neuropsychological battery, the following were administered at baseline, 6, and 12 months: Direct Assessment of Functional Status–Revised (DAFS-R) [58], Number-letter computer task, Memory toolbox task [59], Geriatric Anxiety Inventory [60], and Apathy Inventory (participant version) [61]. The Beck Anxiety Inventory [62] and Beck Depression Inventory-II [63] were administered at baseline only.

Follow-up testing also included a selection of tests that were administered to participants during the CCNA–COMPASS-ND baseline assessment, and these data were used to determine pre-post cognitive changes. These tests include the Jessen questions, MoCA, Rey Auditory Verbal Learning Test (RAVLT)–immediate and delayed recall [64], Trail Making Test [65], Digit Symbol Substitution test [41], Face-Name Association test–immediate and delayed recall (adapted from a task being used in the CIMA-Q study, www.cima-q.ca/en/home), DKEFS Color Word Interference [66], Geriatric Depression Scale [67], Activities Specific Balance Confidence Scale [68], Pittsburg Sleep Quality Index [69], MAYO clinic fluctuations scale, and Quality of Life–AD scale.

Remote cognitive assessments during COVID-19 were conducted via the Zoom video conferencing application. Tests were administered by a trained assessor and participants were asked to set up their computer/camera in a quiet room without any distractions. A package with all testing forms and a return envelope was mailed to participants who were instructed not to open the package until the beginning of the scheduled session. The assessor helped to work out any technical issues and made sure the participant and the test forms were in view of the camera. All the follow up tests listed above were administered except for DAFS-R, Number-letter computer task, and Face-Name Association Task. At the end of the assessment, the participants were instructed to seal the testing forms in the return envelope and mail it back.

#### Blood samples

Fasting blood samples were collected and analyzed for inflammatory cytokines, oxidative stress, and BDNF and APOE status. Baseline and follow-up blood draws were done at the same memory clinic and were drawn according to COMPASS-ND protocols. An additional tube for vitamin K analysis was collected at all time points as an indicator of a healthy diet [70]. Vitamin K was assessed by high-performance liquid chromatography (HPLC) [71]. HbA1c and vitamin K were processed, stored, and analyzed locally. Broader measures of biological markers (e.g., LDL, HDL, triglycerides, etc.) were drawn and stored by the CCNA, but were not analyzed by the time this manuscript was prepared.

#### Diet

Dietary change was assessed using the Canadian Diet History Questionnaire II (C-DHQ II) [51]. The C-DHQ II is a web-based, 153-item questionnaire that takes approximately 1 h to complete. Participants completed the questionnaire during the first and last group session so that the dietitians were available to assist. Items from the C-DHQ II were mapped on to items from the Brain Health Food Guide and target ranges for 14 key foods were used to assess dietary adherence. For “foods to include,” we rated participants’ adherence to each of these items as either 1 = greater than or equal to the target, 0.5 = intake greater than or equal to 50% of the target, 0 = below 50% of the target. For “foods to limit,” we rated participant’s adherence as either 1 = intake less than or equal to the target, 0.5 = intake exceeding the target by an amount less than or equal to 50% of the target, or 0 = intake exceeding the target by an amount greater than 50% of the target. These ratings were summed to calculate a composite Brain-healthy Eating Index (BEI) score representing a participant’s adherence to the study diet (maximum scores = 14).

#### Fitness (V̇O_2peak_)

To assess fitness changes and exercise compliance, participants underwent graded exercise tests at baseline and 6 months using a cycle ergometer (Ergoline 800 P) or a treadmill (Quinton®) depending on balance, mobility, and participant preference. Participants were tested using the same modality at baseline and follow-up. The Bruce protocol was used for treadmill testing [72] and for the cycle-ergometer protocol, the workload was increased by 50 kpm per minute [54]. A 12-lead electrocardiogram (Quinton®, Q-Stress system) was monitored continuously and breath-by-breath gas samples were collected and averaged over 20-s periods via calibrated metabolic cart (VMAX Encore and Spectra – CareFusion, Yorba Linda, California, USA). Peak oxygen consumption (V̇O_2peak_) was determined.

### Analysis

We performed statistical analyses using R (v.3.3.1) [73]. Demographic data are presented as means and standard deviations or frequencies/percentages where appropriate. Frequencies/percentages were used to summarize recruitment, retention, and adherence. Responses to quantitative questions from the surveys were summed and reported. For open-ended questions, responses from 2 or more participants with similar themes were grouped together and reported.

Descriptive statistics (mean and SD) were used to describe within-group changes from baseline to 6 months for outcomes included in our shortened post-intervention assessment. Between-group differences in mean change with confidence intervals and effect sizes were reported for each measure. Effect sizes (Cohen’s *d*) were calculated for within-group change from baseline to 6 months (mean change/pooled SD) and the difference in effect sizes between groups was reported.

## Results

### Participants

**Fig. 1:**
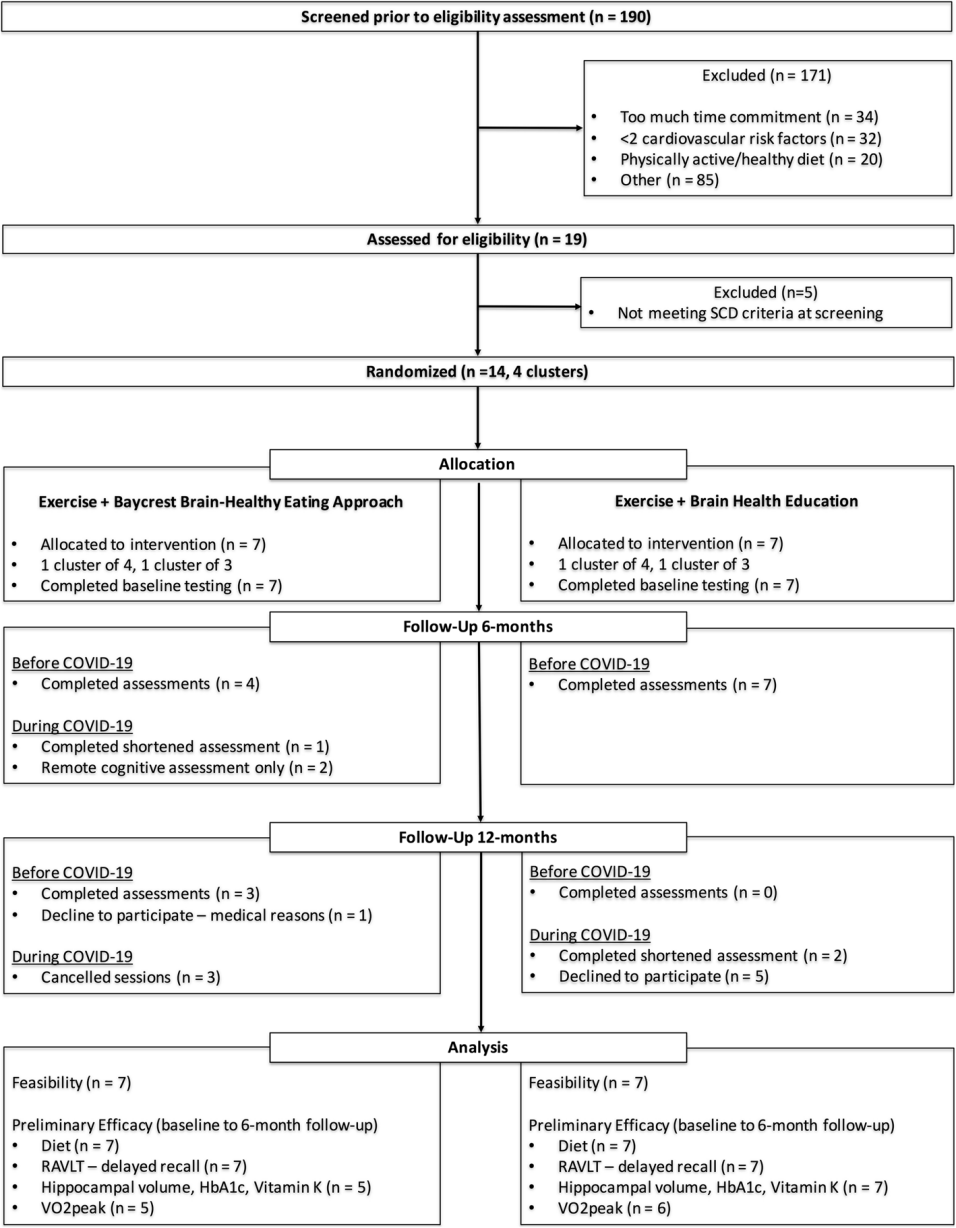
Study flow diagram

A total of 14 participants were cluster randomized in groups of 3–4 to DIET (*n* = 7) or ED (*n* = 7) (Fig. 1). Participant baseline characteristics are displayed in Table 2. The mean age of our sample was 72 ± 5 years and included 10 (71%) females. Average years of formal education were higher in the DIET group (18.0 versus 13.4 years). The difference was driven by one participant with 23 years of education in the DIET group and one participant with 8 years of education in the ED group. All other measures were comparable between study groups.

**Table 2:** Participant characteristics at baseline

Demographics	DIET (***n*** = 7)	ED (***n*** = 7)
Age (years)	72 (5)	71 (6)
Sex	4 females (57%)	6 females (86%)
Education (years)	18 (2.5)	13 (2.7)
Early MCI	1 (14%)	2 (29%)
MoCA (out of 30)	27.4 (3.1)	26.3 (3.1)
Body Mass Index (kg/m^2^)	29.0 (5.0)	30.1 (5.1)
Overweight (*n*)	5 (71%)	6 (86%)
Hypertension (*n*)	5 (71%)	4 (57%)
High Cholesterol (*n*)	4 (57%)	4 (57%)
Pre-diabetes (*n*)	3 (43%)	0 (0%)
Type 2 Diabetes (*n*)	1 (14%)	1 (14%)
V̇O_2peak_ (mL kg^−1^ min^−1^)	22.16 (5.35)^a^	20.13 (3.64)^b^
BEI diet score	6.43 (1.06)	7.21 (1.60)
Hippocampal volume (mm^3^)	3336 (557)^a^	3156 (262)
RAVLT scores (out of 15)	9.00 (1.91)	9.86 (2.27)
HbA1c (%)	6.00 (.29)^a^	5.76 (.57)
Serum vitamin K (nmol/L)	1.24 (0.39)^a^	2.65 (1.61)

All data are reported in mean (SD) or frequency (%). *MCI* mild cognitive impairment, *MoCA* Montreal Cognitive Assessment, *V̇O*_*2peak*_ peak oxygen uptake, *HbA1c* hemoglobin A1c, *BEI* Brain-healthy Eating Index. Overweight = BMI > 25; hypertension, high cholesterol and type 2 diabetes were physician diagnosed

^a^5 participants

^b^6 participants. Participant feedback

### Feasibility

#### Recruitment

Recruitment started in July 2018 and ended when the trial was discontinued in March 2020. Of the 190 people screened, 14 (7%) were enrolled in the trial constituting 21% of our target enrollment (Fig. 1). This resulted in smaller intervention groups than originally planned. The main reasons for not participating were (1) too much time commitment (18%), (2) not meeting criteria for vascular risk factors (17%), or (3) not meeting the criteria for being physically inactive or having a poor diet (11%). Of the 14 enrolled participants, 5 (36%) responded to newspaper advertisements, 4 (29%) responded to community advertisements/flyers, and 3 (21%) were recruited following public talks given by the study investigators. No participants were recruited through the Baycrest participant database, study memory clinics, or the Cardiovascular Prevention and Rehabilitation Program.

#### Retention

All 14 participants completed the intervention. All 6-month assessment visits before COVID-19 were completed (11 assessments) (Fig. 1). Out of 3 participants scheduled to attend their 6-month assessment during COVID-19, 1 completed the shortened assessment while the other 2 only participated in the remote cognitive and diet assessments. One participant was unable to set up video conferencing so the RAVLT was administered over the telephone and is the only cognitive test for which we had complete data at 6 months.

Out of 4 participants who were scheduled for their 12-month follow-up assessment before COVID-19, 3 completed the assessment and one declined to attend due to medical reasons (Fig. 1). Out of 7 participants that were scheduled for follow-up assessments during COVID-19, 2 completed the shortened assessments and 5 declined to participate. Future 12-month assessments for 3 participants were canceled when the trial was discontinued.

#### Adherence

In the DIET group, mean exercise class and DIET session attendance was 90% and 92%, respectively. In the ED group, mean exercise class and ED session attendance was 86% and 91%, respectively. COVID-19 restrictions required us to supplement the once weekly in-person exercise sessions with at-home exercise during the last 6 weeks of the trial for our final DIET cohort (*n* = 3). Study staff followed up with participants on the same day every week to collect exercise information and discuss any questions/concerns with participants. The once-weekly DIET sessions were moved online and over Zoom video conferencing with all participants (*n* = 3) and the dietitian in attendance. Attending the exercise follow-up calls and the group Zoom video conferencing sessions were considered as attending class for analysis purposes.

Participants in the DIET group completed all of their monthly diet self-assessments (100%). Mean exercise log completion was 61% in the DIET group and 39% in the ED group. When attending weekly in-person exercise sessions, participants verbally confirmed they were completing their exercises at home which was evident by progression of their exercise prescriptions over time. Within the DIET group, 5 participants scheduled 3 individual meetings with the study dietitian and 2 participants scheduled 1 session. In the ED group, 2 participants scheduled 2 sessions with the study dietitian and 5 participants scheduled 1 session.

#### Safety

No serious adverse events were reported. Several participants with pre-existing osteoarthritis occasionally complained of mild-moderate discomfort associated with exercise. One participant in the DIET group experienced knee pain resulting from the consecutive 6-min walk test and 5 times sit to stand test. This prevented the individual from participating in intense exercise for 2 weeks, restricting them to light walking and cycling. The aforementioned fitness assessments were not included in our shortened assessment during COVID-19 and therefore were not explored as preliminary efficacy outcomes.

#### Participant feedback

Table 3 provides detailed information on participant feedback. All participants felt that the exercise classes and DIET/ED sessions provided them with new or useful knowledge. In particular, participants in the DIET intervention felt that they were given adequate information to follow the Brain Health Food Guide and that the goal setting/strategy approach helped them make dietary changes. The majority of participants (71%) found the length of the exercise and DIET/ED sessions to be adequate and all participants (100%) stated that they would recommend the study to others who were concerned about their memory.

**Table 3:** Participant feedback

Questions	Responses
**Exercise feedback (*n*** **= 14)**	
**Did the exercise class provide you with new skills or useful knowledge?**	Yes = 14 No = 0
**Were you given adequate resources to continue your exercise routine outside of in-person sessions?**	Yes = 14 No = 0
**What part of the sessions did you find most interesting/useful?**	● Receiving individualized exercise prescription (4) ● Learning to monitor heart rate (3) ● Attending exercise education (3) ● Utilizing the walking track (1) ● Access to exercise equipment at TRI (1) ● Receiving proper instruction/demonstration (1) ● Learning resistance training (1) ● Progress assessments and feedback (1) ● Staff encouragement (1)
**Which part of the sessions did you find least interesting/useful?**	● Resistance training (1) ● Some of the education lectures (1) ● Other members in class not having comparable fitness levels (1)
**What were some of the challenges following the exercise program?**	● Maintaining a regular schedule / exercising daily (3) ● Keeping up with resistance training (2) ● Adjusting program to cater to health issues (2) ● Slowing down and not over exercising (2) ● Pain after exercise (1) ● Doing exercises in winter (1) ● Exercising consistently during the summer, best time for gardening (1) ● The rigor of the baseline evaluation and required workouts aggravated my OA (1) ● Increasing heart rate (1) ● Exercising [at home] without gym equipment (1)
**DIET feedback (*n*** **= 7)**	
**Did the sessions provide you with new skills or useful knowledge?**	Yes = 7 No = 0
**Did the goal setting approach help you make dietary changes?**	Yes = 7 No = 0
**Were you provided with adequate information resources to follow the study diet?**	Yes = 7 No = 0
**The length of the weekly sessions was…**	Adequate = 4 Too long = 1 Too short = 2
**The number of individual sessions with the dietitian were…**	Adequate = 6 Too few = 1 Too many = 0
**Would you recommend this intervention to other concerned about their memory?**	Yes = 7 No = 0
**Which part of the sessions did you find most interesting/useful?**	● Emphasis on fruits and nuts (2) ● Gradual change (1) ● Limited goals each week (1) ● The cooking handouts (1) ● Learning foods to limit (1) ● The importance of oils (1) ● Good input and questions from fellow participants (1) ● Sharing information/resources with each other (1)
**Which part of the sessions did you find least interesting/useful?**	● The recipes and cooking advice (1) ● Need more info on switching from meat eater to vegetable eater (1) ● Clarification on healthy food myths (1)
**What were some of your challenges following the study diet?**	● Eating the required fruits and vegetables (2) ● Reducing sugar, butter, and meat (1) ● Meal planning and meeting my personal goals (1) ● Making sure the required groceries were available (especially difficult during COVID restrictions) (1) ● Following recipes (1) ● IT difficulties during remote sessions (1) ● Avoiding highly processed foods (1)
**If this intervention is repeated, what should be left out, added, or changed?**	● More info on the process/psych of change and goal setting (2) ● More emphasis on the scope of change contemplated (1) ● More incorporation of individual cultural foods (1) ● Wish it would move faster (1)
**ED Feedback (*n*** **= 7)**	
**Did the sessions provide you with new skills or useful knowledge?**	Yes = 7 No = 0
**The length of the weekly sessions was…**	Adequate = 6 Too long = 1 Too short = 0
**The number of individual sessions with the dietitian were…**	Adequate = 6 Too few = 1 Too many = 0
**Would you recommend this intervention to others concerned about their memory?**	Yes = 7 No = 0
**Which part of the sessions did you find most interesting/useful?**	● Information on sleep (2) ● Talks about how seniors can improve/maintain their memory (1) ● Procedural memory sessions (1) ● Provoked curiosity to learn more (1) ● Lectures about the brain [anatomy] (1)
**Which part of the sessions did you find least interesting/useful?**	● Technical information about the brain (1) ● Sessions on different types of memory (1)
**If this intervention is repeated, what should be left out, added, or changed?**	● Leave out the review session (1) ● Add specific exercises one can do to improve brain functioning/memory (1) ● Add emerging brain health knowledge/information (1)

### Preliminary efficacy

#### Hippocampal volume

A total of 5 DIET and 7 ED individuals participated in the MRI assessment at baseline and 6 months. Mean unadjusted hippocampal volume at baseline was 3336.16 ± 557.26 mm^3^ in the DIET group and 3155.94 ± 262.03 mm^3^ in the ED group. At 6 months, mean hippocampal volumes were 3337.95 ± 506.18 mm^3^ in the DIET group and 3165.69 ± 290.83 mm^3^ in the ED group. A negligible between-group effect was observed (*d* = .11) (Table 4).

**Table 4:** Preliminary efficacy results

**Outcome**	***n***	**Baseline**	**6 months**	**Mean Change**	**95% Confidence Interval**	***d***
**Hippocampal volume** **(mm**^**3**^**)**						
DIET	5	3336 ± 557	3338 ± 506	2 ± 139		
ED	7	3156 ± 262	3166 ± 291	10 ± 90		
Between group difference				8	(-164 to 179)	.11
**RAVLT**						
DIET	7	9.00 ± 1.91	10.57 ± 2.51	1.57 ± 3.10		
ED	7	9.86 ± 2.27	10.57 ± 2.15	.71 ± 1.50		
Between group difference				.86	(-2.11 to 3.82)	.35
**HbA1c (%)**						
DIET	5	6.00 ± 0.29	5.52 ± 0.18	-.48 ± .19		
ED	7	5.76 ± 0.57	5.71 ± 0.55	-.05 ± .05		
Between group difference				.43	(.20 to .67)	1.02
**Vitamin K (nmol/L)**						
DIET	5	1.24 ± 0.39	1.45 ± 1.24	.21 ± .88		
ED	7	2.65 ± 1.61	1.93 ± 0.98	-.90 ± 1.25		
Between group difference				1.11	(-.26 to 2.48)	.70
**BEI score (out of 14)**						
DIET	7	6.43 ± 1.06	9.36 ± 1.28	2.93 ± 1.13		
ED	7	7.21 ± 1.60	7.07 ± 1.67	-.14 ± 2.19		
Between group difference				3.07	(.96 to 5.18)	1.75
**V̇O**_**2peak**_ **(ml/kg/min)**						
DIET	5	22.16 ± 5.35	24.20 ± 5.82	2.04 ± .88		
ED	6	20.13 ± 3.64	22.53 ± 3.23	2.40 ± 2.81		
Between group difference				.36	(-3.31 to 2.59)	.17

#### RAVLT–delayed recall

Our analysis of cognitive function was limited to RAVLT scores as this was the only cognitive test with a full data set. These data included 3 remote assessments (2 video conference, 1 telephone) conducted at 6-months follow-up in the DIET group. Mean RAVLT delayed recall scores at baseline were 9.00 ± 1.91 in the DIET group and 9.86 ± 2.27 in the ED group. At 6 months, mean scores were 10.57 ± 2.51 in the DIET group and 10.57 ± 2.15 in the ED group. There was a small between group-effect (*d* = .35) indicating greater improvement in the DIET group (Table 4).

#### HbA1c

A total of 5 DIET and 7 ED individuals participated in a fasting blood draw at baseline and 6 months. Mean HbA1c values at baseline were 6.00 ± 0.29% in the DIET group and 5.76 ± 0.57% in the ED group. At 6 months, mean HbA1c levels were 5.52 ± 0.18% in the DIET group and 5.78 ± 0.57% in the ED group. There was a large between-group effect (*d* = 1.02) indicating a greater reduction in the DIET group. Additionally, all 4 of the participants in the DIET group who were in the pre-diabetic or diabetic range (HbA1c ≥ 6.0%) at baseline moved into the normal range (HbA1c < 6.0%) (Table 4).

#### Vitamin K

Mean serum vitamin K levels at baseline were 1.24 ± 0.39 nmol/L in the DIET group (*n* = 5) and 2.65 ± 1.61 nmol/L in the ED group (*n* = 7). At 6 months, mean vitamin K levels were 1.45 ± 1.24 nmol/L in the DIET group and 1.93 ± 0.98 nmol/L in the ED group. There was a medium sized between-group effect (*d* = .70) indicating greater improvements in the DIET group (Table 4).

#### Brain-healthy Eating Index score

**Fig. 2:**
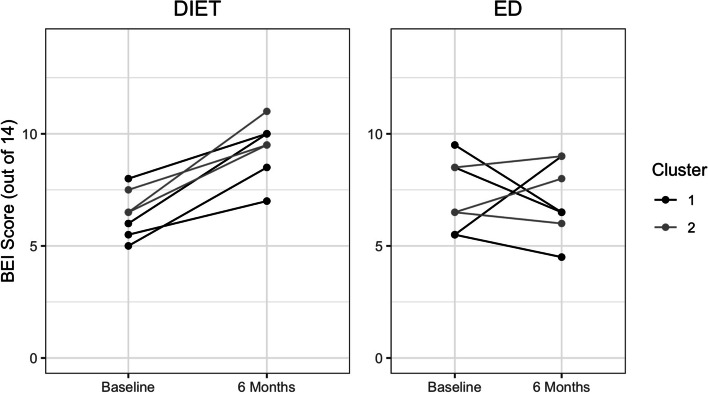
Individual changes in Brain-healthy Eating Index (BEI) scores by cluster

At baseline, mean Brain-healthy Eating Index (BEI) scores were 6.43 ± 1.06 in the DIET group and 7.21 ± 1.60 in the ED group. The most common food items outside target ranges at baseline were raw leafy vegetables (86%), total fruit (86%), and fatty fish (86%). At 6 months, mean BEI scores were 9.36 ± 1.28 in the DIET group and 7.07 ± 1.67 in the ED group. There was a large between-group effect (*d =* 1.75) indicating greater dietary change in the DIET group (Fig. 2). The most common food items that were improved within the DIET group were cruciferous vegetables (33%) and fatty fish (25%). All participants whose cruciferous vegetable or total meat and poultry intake was outside-target at baseline improved to within-target ranges at post-intervention.

#### V̇O_2peak_

A total of 5 DIET and 6 ED individuals completed the graded exercise test at baseline and 6 months. One participant in the ED group attended the 6-month assessment but did not complete their exercise test due to difficulties with the mouthpiece. Mean V̇O_2peak_ values at baseline were 22.16 ± 5.35 mL kg^−1^ min^−1^ in the DIET group and 20.13 ± 3.64 mL kg^−1^ min^−1^ in the ED group. At 6 months, mean V̇O_2peak_ values were 24.2 ± 5.82 mL kg^−1^ min^−1^ in the DIET group and 22.53 ± 3.23 mL kg^−1^ min^−1^ in the ED group. There was a negligible between-group effect (*d =* .17) (Table 4).

## Discussion

This study investigated the feasibility and preliminary efficacy of the LEAD trial. All participants completed the trial and attended 90% of exercise and diet/education visits on average. No serious adverse events occurred and all participants expressed positive feedback about the intervention. Recruitment difficulties owing to the perceived time commitment and narrow inclusion criteria resulted in a smaller than anticipated sample size. Preliminary findings indicate large improvements in diet quality and clinically significant decreases in HbA1c levels among DIET participants compared to ED. We conclude that the LEAD trial is feasible; however, different recruitment strategies or location/method of delivery would be needed to meet realistic sample sizes required to assess efficacy.

### Feasibility

Recruitment into the LEAD trial was challenging. The majority of our participants were recruited from newspaper and community advertisements, and talks by our investigators. Recruiting through clinical practices proved not to be feasible. Compared to individuals with greater cognitive impairment, those with SCD or early MCI are less likely to seek help and be referred to memory clinics, and the few individuals with SCD or early MCI that were referred to us from these sources did not meet our other criteria (i.e., risk factors, diet, exercise). Several patients were screened at the Cardiovascular Prevention and Rehabilitation program, but the time needed to complete their baseline assessments and organize an intervention group would have delayed their entry into the program beyond an appropriate time frame. By shortening assessment frequency and duration, this line of recruitment may be more feasible.

Most people at initial contact expressed that the once weekly in-person visits and the required assessments was too much of a time commitment. Travel to the study sites was deemed challenging for some. The requirement to be enrolled and complete assessments for both LEAD and COMPASS-ND may have been a deterrent for potential participants. Expanding eligibility criteria to include other vascular risk factors associated with cognitive decline may also help with recruitment, for example, smoking, transient ischemic attack, atrial fibrillation, and sleep apnea [74–77]. It may also be appropriate to include individuals with only one vascular risk factor in addition to low physical activity and poor diet.

We attribute high retention and adherence to the close contact we kept with participants. Our research staff were always available to discuss any questions or concerns and participants were sent out appointment schedules and reminders about upcoming assessments. If a drop in attendance was noticed, the participant was contacted to discuss the reasons and/or barriers and help find solutions. Participants (*n* = 3) were satisfied with the video conferencing sessions conducted during the last 6 weeks of the trial. Attendance rates were the same as in-person sessions and there were minor technical difficulties. High assessment completion rates seen in our sample before the pandemic (100%) indicate that our extensive testing battery was manageable for participants. The reported adverse event related to consecutive fitness assessments indicates that for older adults, especially those with chronic musculoskeletal conditions, it may be necessary to take breaks between strenuous physical assessments.

Participants in the DIET group filled out 100% of monthly diet self-assessment and 61% of weekly exercise logs compared to 39% of exercise logs in the ED group. Diet self-assessments were required less frequently and usually completed with the help of the study dietitian which may explain the discrepancy between exercise and diet reporting. This may also speak to the ability of the DIET delivery method to motivate participants to track their behaviors which may have contributed to increased exercise reporting in the DIET group. Participants verbally confirmed that they were completing their exercises at home, but either forgot to fill out the logs or found them too onerous. Fitness changes seen in our sample support these claims. Objective measures such as pedometers or accelerometers are ways to overcome these challenges; however, full time monitoring can be expensive, burdensome, and require greater staff involvement for support and analysis. Another strategy would be to have more supervision of the intervention where objective accounts of exercise participation could be recorded.

### Preliminary efficacy

We attribute the promising dietary changes associated with the DIET intervention to our novel form of delivery which combined dietary education with guiding participants to set, monitor, and track their diet related goals. The dietary changes observed in LEAD are comparable to high-quality interventions of brain healthy global diets such as Mediterranean, MIND, and DASH diets [28, 46, 47]. The DIET group increased their dietary adherence from an average 46% at baseline to 67% at 6 months, an absolute mean increase of 21%. In PREDIMED, diet adherence increased by ~ 25% following a 12-month intervention of a Mediterranean diet supplemented with either mixed nuts (30 g/day) or extra virgin olive oil (1 L/week) [47]. In the 4-month ENCORE trial, adherence to the DASH diet increased by 30% and 22% in groups receiving DASH diet + weight management and DASH diet alone, respectively [46]. In the Nu-AGE trial, the intervention group increased their adherence to a Mediterranean-type diet by an average of 14% from 51.9% at baseline to 65.9% at 1-year follow-up [28]. Dietary change as indicated in this study provides proof-of-concept evidence that DIET is effective at improving diet quality in this population.

While several high-quality diet interventions have observed associations between diet and brain changes, we were not powered to corroborate these findings. We only achieved 21% of the sample size estimated to observe between group differences in hippocampal volume change. The small to moderate between-group effect size observed for changes in cognitive performance is a promising preliminary finding; however, a larger study is required to statistically analyze cognitive differences associated with dietary intervention.

The large between-group effect in HbA1c change represents a clinically meaningful reduction in the DIET group that is associated with a decreased risk of cardiovascular disease [78]. Reducing HbA1c levels has also shown to be associated with a decreased risk of incident all cause dementia and Alzheimer’s disease [79]. It would be worthwhile to further investigate whether reductions in HbA1c associated with the DIET intervention are associated with cognitive changes.

A moderate between-group effect for change in serum vitamin K levels was observed. Green leafy and cruciferous vegetables are an important food source of vitamin K, and its levels in serum can provide information about dietary patterns [80]. Reduced vitamin K status has also been associated with poor cognitive function in older adults [71, 81]. Although vegetable intake was a commonly altered component of diet, improvements in the DIET group also resulted from other aspects of diet such as increasing fatty fish consumption, or decreasing processed foods.

Lastly, large improvements in fitness were observed in both study arms, and while it seems logical that this was due to the intervention, a proper control group is needed. There were no differences between groups in our study. It would be interesting to investigate in a larger trial whether the goal setting and strategy skills developed in DIET transfer to increased physical activity levels.

### Limitations, strengths, and future considerations

A major limitation to our study design was the lack of a control group that did not participate in exercise. This prevents us from determining the effects of exercise on our outcome measures. We were, however, able to demonstrate the feasibility of the exercise program as part of the LEAD trial. We had originally planned to compare ED to a stretching program with a similar population in a collaborator’s study, but given that they had more significant vascular impairment we deemed this analysis to be inappropriate. Typically, exercise interventions have shown small to moderate effects on cognition compared to control [20].

Another limitation is that our study diet relied on evidence demonstrating that the PREDIMED diet was associated with cognitive changes [25] in older adults. We also compare adherence rates in our study with those of PREDIMED. A major flaw of PREDIMED that was discovered after publication was the improper randomization of approximately 20% of participants. While cognitive changes were only assessed in a subset of these participants, it is uncertain how randomization bias affected these results. Concerns remain about other details of the trial; however, this is still the largest and most influential trial in the field and contributes to our understanding of the relationship between Mediterranean diet and cognition.

Slow recruitment resulted in a much smaller than anticipated sample size. The DIET program was designed to accommodate groups of 4–6 participants, but we ended up running groups of 3–4. It is unknown if adherence rates would differ based on group size; however, groups of 4–6 were considered ideal based on prior work [50] and we feel that these concerns may be minimal. Dietary changes and positive feedback received from participants suggest that the intervention was feasible with smaller groups. The small sample size prevented analyses at the cluster level, but in an effort to highlight potential cluster effects, we have displayed our data by cluster in Fig. 2. Unlike typical cluster randomized designs, all our clusters were drawn from the community so we would not expect the same variability as with other designs. We were also not powered to examine gender differences among our sample. This is an important consideration for a larger trial, as there is evidence for gender differences in exercise and dietary preferences and adherence rates [82, 83].

The arrival of COVID-19 greatly impacted the LEAD trial. We abruptly had to cancel follow-up assessments that were scheduled and put an indefinite pause on recruitment. When our assessment sites approved the restart of research activities, several participants declined to attend which resulted in incomplete data collection. We also conducted an abridged remote cognitive assessment for several participants in the DIET group. It is likely that they performed worse when being assessed remotely compared to being in the direct presence of an assessor. Standardized forms of remote testing are becoming more necessary and may be more readily available for future trials.

Strengths of this study include the adoption of a brain-healthy diet based on empirical evidence and the use of a counseling approach that teaches goal setting strategies to achieve sustainable dietary change. We also created a composite score called the Brain-healthy Eating Index to assess dietary adherence utilizing a common diet assessment tool. Considering that the online diet counseling sessions appeared to be feasible within our study, one avenue to explore would be delivering DIET intervention remotely. Technology use has rapidly increased among older adults during the COVID-19 pandemic and is expected to dramatically increase in the coming years [84, 85]. Online intervention delivery and assessment would allow studies to cast a wider net for recruitment and could also avoid potential future disruptions of face to face contact like we have seen with the COVID-19 pandemic.

## Conclusions

High adherence and retention rates were observed among LEAD participants. Preliminary findings illustrate improvements in diet quality and HbA1c. We attribute successful dietary adherence to the novel form of intervention delivery which combines diet education with cognitive behavioral strategies. The results of this study indicate that the trial interventions are feasible if difficulties surrounding recruitment can be mitigated.

## Supplementary Information

**Additional file 1:.** CONSORT 2010 checklist of information to include when reporting a pilot or feasibility trial.

**Additional file 2: Appendix 1.** COMPASS-ND procedures by visit. **Appendix 2.** Diet Screening Questionnaire. **Appendix 3.** Brain Health Food Guide pamphlet. **Appendix 4.** Example DIET and ED group class schedule. **Appendix 5.** Eating Plan Self-Assessment. **Appendix 6.** Exercise session feedback form. **Appendix 7.** DIET session feedback form. **Appendix 8.** ED session feedback form. **Appendix 9.** Full list of LEAD outcome assessments.

## Data Availability

The data was uploaded to the CCNA LORIS database. Access to the data will be subjected to the CCNA data access policy. No datasets are included in this this manuscript. Trial registration The LEAD study is registered with the US National Institutes of Health clinical trials registry (ClinicalTrials.gov identifier NCT03056508) and this report complies with the Standard Protocol Items: Recommendations for Interventional Trials (SPIRIT) statement.
