# Supplementary material for: Feasibility and preliminary efficacy of the LEAD trial: a cluster randomized controlled lifestyle intervention to improve hippocampal volume in older adults at-risk for dementia
Source: Pilot Feasibility Stud. 2022 Feb 9;8:37. doi: 10.1186/s40814-022-00977-6 (PMC8826667; doi:10.1186/s40814-022-00977-6)
Supplement: Supplementary file 2 — Additional file 2: Appendix 1. COMPASS-ND procedures by visit. Appendix 2. Diet Screening Questionnaire. Appendix 3. Brain Health Food Guide pamphlet. Appendix 4. Example DIET and ED group class schedule. Appendix 5. Eating Plan Self-Assessment. Appendix 6. Exercise session feedback form. Appendix 7. DIET session feedback form. Appendix 8. ED session feedback form. Appendix 9. Full list of LEAD outcome assessments. [file 40814_2022_977_MOESM2_ESM.docx]

**Additional File 2**

**Appendix 1: COMPASS-ND procedures by visit**

**Visit 1: Screening and demographics (2-2.5 hours)**

- Written informed consent prior to study procedures (study investigator or delegate of investigator; participant and study partner)
- Assess inclusion/exclusion criteria (site staff; participant and study partner)
- Audiometry (site staff; participant)
- MoCA (all groups. SCI must have a score ≥ 25; all participants must have a score >13) (site staff; participant)
- Subjective Memory Assessment (SCI only) (site staff; participant)
- Logical Memory 1 & 2 from Wechsler memory scale (for SCI, MCI, V-MCI, AD, Mixed) (psychometrician; participant)
- Sociodemographic Data
- Benson Figure Recall (for FTD, PD) (psychometrician; participant)
- Physical activity questionnaires
- CERAD word list Recall (for SCI, MCI, V-MCI, AD, Mixed) (psychometrician; participant)
- Lawton Brody IADL scale (for SCI, MCI, & Vascular MCI) (site staff; study partner)
- GDS 30
- GAD 7
- Clinical Dementia Rating scale (for SCI, MCI, V-MCI) (site staff; study partner and participant)
- Clinical PPA and bvFTD features from NACC Uniform Data Set FTLD Module (FTD) (physician or nurse; study partner)
- Research Diagnosis
- Provide participant with take-home assessment packet:
  - Hobbies and leisure activities (participant or study partner if the local investigator or staff determines that the participant is unable to complete the questionnaire)
  - Tobacco and alcohol consumption (participant or study partner if the local investigator or staff determines that the participant is unable to complete the questionnaire)
  - Activities of Daily Living (study partner)
  - Quality of Life (participant)
  - Oral Health (participant or study partner if the local investigator or staff determines that the participant is unable to complete the questionnaire)
  - End of Life Care (participant or study partner if the local investigator or staff determines that the participant is unable to complete the questionnaire)
  - Social network, support, & activities (participant or study partner if the local investigator or staff determines that the participant is unable to complete the questionnaire)
  - Adverse childhood experiences questionnaire (optional) (participant or study partner if the local investigator or staff determines that the participant is unable to complete the questionnaire)
  - Neuropsychiatric Inventory - Questionnaire (study partner)
  - Mild Behavioral Impairment Checklist (study partner)
  - Delirium questionnaire

**Visit 2: Clinical and Physical Assessments (2.5-3.5 hrs.)**

- Fasting blood collection (nurse; participant)
- Saliva collection (nurse; participant)
- Urine collection (nurse; participant)
- Physical Measurements (nurse; participant)
- Health perception, fatigue, falls history & balance assessment (site staff; participant)
- Buccal swab (optional)
- Walking speed (4 and 6m) (site staff; participant)
- Grip strength (site staff; participant)
- Vision assessment (site staff; participant)
- Hearing questionnaire & computer task (site staff; participant)
- Olfaction assessment (site staff; participant)
- Sleep (site staff; participant or study partner)
- Cognitive fluctuations (site staff; study partner)
- Nutrition (site staff, participant)
- Caregiver burden assessment (coordinator; participant [if in a caregiving role])
- Current and past medications (site staff; participant or study partner)
- Medical, mental health, and surgical history (site staff; participant or study partner)
- Family history (site staff; participant or study partner)
- Initial Disease Symptoms (site staff; participant or study partner)
- Disease course (site staff; participant or study partner)
- Signs and Symptoms (physician; participant)
- Physical examination (physician; participant)
- Neurological examination (physician; participant)
- Hachinski Ischemic Scale (physician; participant)
- Clinical Diagnosis (physician)

**Visit 3: Neuropsychological Assessment**

- WAIS-III Vocabulary Test
- Rey Auditory Verbal Learning Test
- Envelope Test
- Trail Making Test A & B
- Birmingham Object Recognition Battery Object Decision Task – Easy B
- D-KEFS Color-Word Interference Test
- Face-Name Association Task
- WAIS-III Digit Symbol – Coding & Incidental Learning
- Sentence Inhibition (Haylings) Task (computer test)
- D-KEFS Category Fluency Test
- D-KEFS Letter Fluency Test
- Brief Visuospatial Memory Test – Revised
- WAIS-III Digit Span Test
- CCNA Reaction Time Task (computer test)
- Social Norms Questionnaire
- Judgment of Line Orientation Test
- Noun and Verb Naming Subtests
- Semantic Word-Picture Matching Test
- Semantic Associates Test
- Northwestern Anagram Test (Short Form)
- Sentence Repetition Test
- Word Reading Test
- Boston Diagnostic Aphasia Exam Cookie Theft Picture Description
- Sentence Reading Test
- Social Behavior Observer Checklist [FTD only]

**Visit 4: MRI scan**

- T1
- PD/T2
- FLAIR
- T2*
- DTI (30 dir.)
- BOLD Resting state

**Visit 5: Lumbar Puncture** (optional)

**Appendix 2: Diet Screening Questionnaire**

**GENERAL QUESTIONS REGARDING DIET AND SUPPLEMENT USE**

1. Would you be willing to try a fiber-rich and low-meat diet with a lot of vegetables, fruits, raw salads, nuts, legumes, and fish? **** YES **** NO **** UNSURE
2. Over the past month, how many alcoholic drinks did you usually have per day? ___________
3. Are you currently eating a special diet or avoiding certain foods for medical (e.g. allergies) or personal reasons (e.g. weight loss)? **** YES **** NO

a. If yes, provide details:____________________________________________________________

b. If yes, would you be willing to stop following this special diet? **€** YES **€** NO

1. Do you consume any supplements such as vitamins, fish oil, or herbal medicines? **** YES **** NO

a. If yes, list the supplements you take

_______________________________________________________________________

b. If yes, would you be willing to stop taking these supplements for the study?

**€** YES **€** NO **€** UNSURE

**DIET QUALITY SCREENING QUESTIONS**

I’m going to ask you about how often you ate certain foods over the past month. I will ask how many times per month, week, or day you ate each food. Feel free to ask questions. Your answers are important. I’ll start with raw greens.

INTERVIEWER NOTE: If a participant responds less than once per month, consider that as “Never”. If participant gives a number without a time frame, ask: “Was that per day, week, or month?” You may help the participant choose an appropriate response based on what they say (i.e. it is not necessary for you to read out aloud each possible option). You may guide struggling participants by initially asking if the food was something they ate a few times a month or week or day. Then narrow down to one of the listed options.

If participant says that they have recently changed their diet, advise them to give their best impression of their usual diet over the last month.

1. Over the past month, how often did you eat **RAW leafy greens**? This category includes lettuce, spinach, mixed greens, kale, cabbage or coleslaw. (1 point if participant eats > 3 serv/wk)

- **Never**
- **1 time in past month**
- **2-3 times in past month**
- **1-2 times per week**
- **3 times per week**
- 4-5 times per week Score = _____
- 6 times per week
- 1 time per day
- 2 or more times per day

1. Over the past month, how often did you eat **peanuts, almonds, walnuts, or other nuts**? Do not include peanut butter. (1 point if participant eats > 3 serv/wk)

- **Never**
- **1 time in past month**
- **2-3 times in past month**
- **1-2 times per week**
- **3 times per week**
- 4-5 times per week Score = _____
- 6 times per week
- 1 time per day
- 2 or more times per day

1. Over the past month, how often did you eat **fatty fish**? This category includes fresh or canned salmon, sardines, herring, and mackerel. Do not include tuna, white fish, shrimp, or any other seafood.

(1 point if participant eats > 1 serv/wk)

- **Never**
- **1 time in past month**
- **2-3 times in past month**
- **1 time per week**
- 2 times per week Score = _____
- 3-4 times per week
- 5-6 times per week
- 1 time per day
- 2 or more times per day

1. Over the past month, how often did you eat **red or processed meat**? This category includes beef, pork, lamb, liver, sausages, hot dogs, cold cuts, jerky, and pepperoni. (1 point if participant eats < 1 serv/wk )

- Never
- 1 time in past month
- 2-3 times in past month Score = _____
- **1 time per week**
- **2 times per week**
- **3-4 times per week**
- **5-6 times per week**
- **1 time per day**
- **2 or more times per day**

5a. Over the past month, how often did you eat **store-bought or commercially prepared**

**baked goods**? This category includes muffins, cookies, scones, donuts, cakes, and pastries.

Do not include bread.

- Never (0 per day)
- 1 time in past month (0.033 per day)
- 2-3 times in past month (0.083 per day)
- 1 time per week (0.14 per day)
- 2 times per week (0.29 per day)
- 3-4 times per week (0.50 per day)
- 5-6 times per week (0.79 per day)
- 1 time per day (1 per day)
- 2 or more times per day (2 per day)

5b. Over the past month, how often did you eat **store-bought or commercially prepared dairy desserts**? This

category includes ice cream, frozen yogurt, milkshakes, puddings, and custards.

- Never (0 per day)
- 1 time in past month (0.033 per day)
- 2-3 times in past month (0.083 per day)
- 1 time per week (0.14 per day)
- 2 times per week (0.29 per day)
- 3-4 times per week (0.50 per day)
- 5-6 times per week (0.79 per day)
- 1 time per day (1 per day)
- 2 or more times per day (2 per day)

**Exclusion criteria based on self-reported diet and supplement use**

*****Participants with 3 or more points must be excluded*****

| **Question** | **Score (1 or 0)** |
| --- | --- |
|  |  |
| 1: Raw leafy greens |  |
| 2: Nuts |  |
| 3: Fatty fish |  |
| 4: Red and processed meat |  |
| 5: Store-bought baked goods and dairy desserts |  |
|  | TOTAL SCORE = |

***Add per day frequencies from questions 5a and 5b together***

***1 point if the sum is < 0.28***

| Question 5a daily frequency ______ + Question 5b daily frequency ______ = ______  Score = _____ |
| --- |

- Food allergy or severe intolerance not compatible with consumption of the intervention diet.
- Unwilling to eat the intervention diet based on the description provided.
- Unwilling to stop following a weight loss diet. Calorie counting is allowed as long as participant is willing to adhere to intervention targets. Discretion will be exercised.
- Unwilling or unable to discontinue consumption of vitamin, mineral, and any other supplements for the duration of the study. Excludes vitamin D and calcium. Discretion will be exercised.
- Unwilling or unable to discontinue a therapeutic diet incompatible with the intervention diet. Includes avoidance of leafy greens, fish, or tree nuts. Discretion will be exercised.

**Appendix 3: Brain Health Food Guide (displayed in pamphlet form)**

**
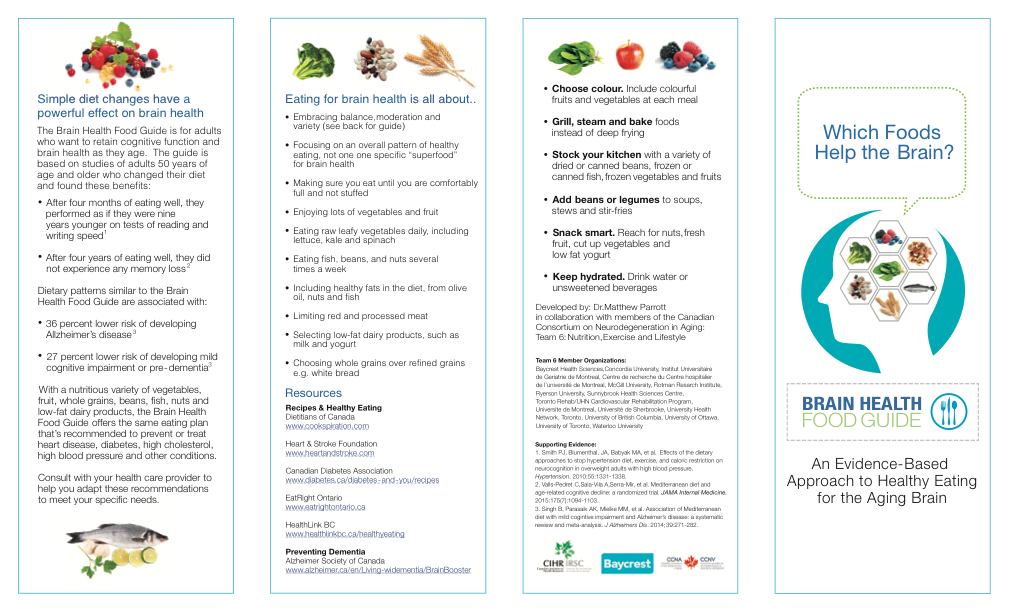
**

**
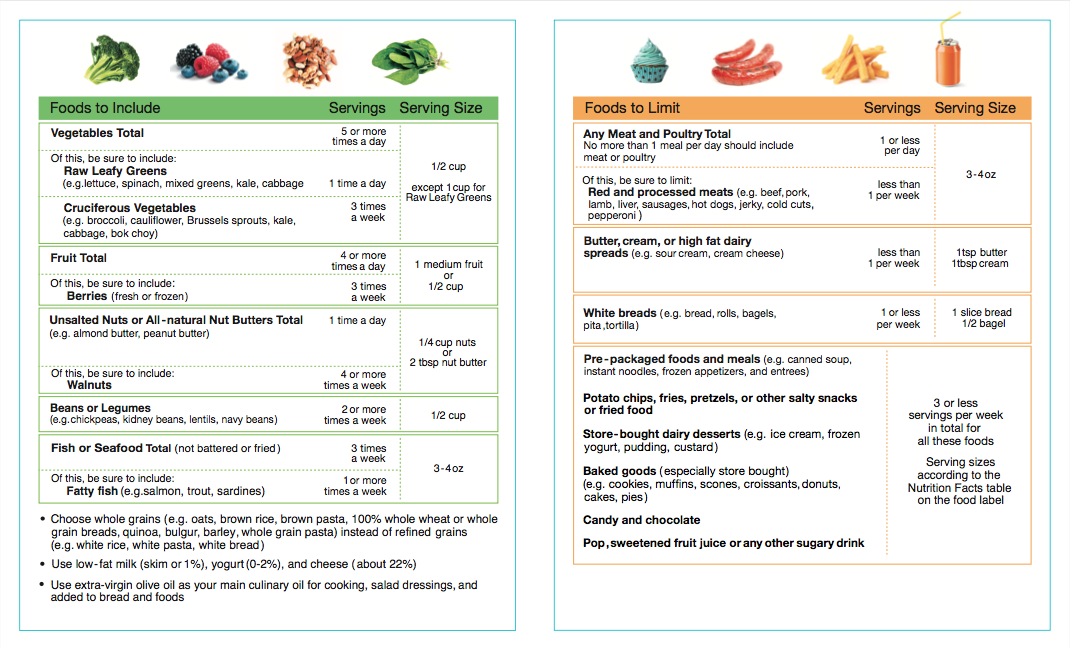
**

**Appendix 4: Example DIET and ED group class schedules**

**DIET Curriculum Outline**

| **Week** | **Date** | **Topic** |
| --- | --- | --- |
| **1** | July 31/18 | Food Frequency Questionnaire Filled Out |
| **2** | Aug 7/18 | Introduction to the Brain Health Food Guide |
| **3** | Aug 14/18 | Introduction to Goal Setting |
| **4** | Aug 21/18 | Vegetables & Fruits ◊ |
| **5** | Aug 28/18 | Healthy Protein |
| **6** | Sep 4/18 | Healthy Fats |
| **7** | Sep 11/18 | Grains |
| **8** | Sep 18/18 | Smart Substitutes/Foods to Limit ◊ |
| **9** | Sep 25/18 | Grocery Shopping Tips/Grocery Shopping Videos |
| **10** | Oct 2/18 | 1:1 Sessions with RD, shorter goal setting session with group |
| **11** | Oct 9/18 | Cardiac Program Fats Lecture; shorter goal setting session with group |
| **12** | Oct 16/18 | Cardiac Program Fibre Lecture; shorter goal setting session with group ◊ |
| **13** | Oct 23/18 | Brainstorming session –Participant ideas on incorporating Brain Healthy Foods |
| **14** | Oct 30/18 | Cardiac Program Label Lecture; shorter goal setting session with group |
| **15** | Nov 6/18 | Cardiac Program Sodium Lecture; shorter goal setting session with group |
| **16** | Nov 13/18 | More about Fish/Nuts/ Vegetables/Recipes/Eating Out or topic chosen by the group ◊ |
| **17** | Nov 20/18 | More about Fish/Nuts/ Vegetables/Recipes/Eating Out or topic chosen by the group |
| **18** | Nov 27/18 | More about Fish/Nuts/ Vegetables/Recipes/Eating Out or a topic chosen by the group |
| **19** | Dec 4/18 | Mindful Eating |
| **20** | Dec 11/18 | Brainstorming session –Brain Healthy Foods During Holidays ◊ |
| **21** | Dec 18/18 | 1:1 Sessions with RD, shorter goal setting session with group |
| **22** | Jan 8/18 | Nutrition Myths, Understanding Media Stories |
| **23** | Jan 15/18 | Sustainable Goals for the Future/Nutrition Resources |
| **24** | Jan 22/18 | Wrap Up/Thank You Session ◊ |

*Note:* ◊ Self Assessment Questionnaire filled in and handed in to RD (once per month). The program was closed on December 25^th^ and January 1^st^

**ED Curriculum Outline**

| **Week** | **Date** | **Topic** |
| --- | --- | --- |
| **1** | May14/19 | Food Frequency Questionnaire Filled Out |
| **2** | May21/19 | Getting to know your brain better |
| **3** | May28/19 | Getting to know your brain better |
| **4** | June4/19 | Movie: Human brain – How smart can we get? |
| **5** | June11/19 | Different types of memory and attention |
| **6** | June18/19 | Different types of memory and attention |
| **7** | June25/19 | Movie: Brain Games |
| **8** | July2/19 | Semantic memory |
| **9** | Sep 25/18 | Episodic memory |
| **10** | Oct 2/18 | Memory and senses |
| **11** | Oct 9/18 | Cardiac Program Fats Lecture * |
| **12** | Oct 16/18 | Procedural memory |
| **13** | Oct 23/18 | Cardiac Program Fibre Lecture* 1:1 dietitian sessions (2 spots) |
| **14** | Oct 30/18 | Cardiac Program Label Lecture* 1:1 dietitian sessions (2 spots) |
| **15** | Nov 6/18 | Cardiac Program Sodium Lecture* |
| **16** | Nov 13/18 | Executive functions |
| **17** | Nov 20/18 | Executive functions |
| **18** | Nov 27/18 | Neuroplasticity |
| **19** | Dec 4/18 | Movie: CBC – While you were sleeping |
| **20** | Dec 11/18 | Sleep and stress management |
| **21** | Dec 18/18 | 1:1 dietitian sessions (4 spots)* |
| **22** | Jan 8/18 | Monitoring health and keeping morale |
| **23** | Jan 15/18 | Food Frequency Questionnaire |
| **24** | Jan 22/18 | Jeopardy review |

*Note:* No group sessions on days when participants will be attending a cardiac program nutrition talk or meeting with a dietitian

**Appendix 5: Eating Pattern Self-Assessment**

STUDY ID: _________________ DATE:__________________

Please think about what you usually ate or drank **over the past month**, that is, the past 30 days. Check the box that best represents your answer. Consult the attached “Serving Size” information sheet if needed.

1. How many servings of **vegetables** did you usually eat?

€ < 1 per day € 1 per day € 2 per day € 3 per day € 4 per day € 5 per day € 6 or more per day

1a. Of the vegetables that you ate, how many servings of **raw leafy greens** did you usually eat? *(e.g.*

*lettuce, spinach, cabbage, kale, mixed greens)*

€ < 1 per week € 1 per week € 2-3 per week € 4-6 per week € 1 per day € 2 or more per day

1b. Of the vegetables that you ate, how many servings of **cruciferous vegetables** did you eat? *(e.g. broccoli,*

*cauliflower, Brussel sprouts, radish, kale, cabbage, turnip, bok choy)*

€ < 1 per week € 1-2 per week € 3 per week € 4-6 per week € 1 per day € 2 or more per day

1. How many servings of **fruit** did you usually consume? *(fresh, frozen, dried, 100% juice)*

 < 1 per day  1 per day  2 per day  3 per day  4 per day  5 per day  6 or more per day

2a. Of the fruit that you ate, how many servings of **berries** did you eat? *(fresh or frozen)*

€ < 1 per week € 1-2 per week € 3 per week € 4-6 per week € 1 per day € 2 or more per day

3. How many servings of **unsalted nuts or all-natural nut butters** (including peanuts) did you eat?

€ < 1 per week € 1 per week € 2-3 per week € 4-6 per week € 1 per day € 2 or more per day

3a. Of the nuts that you ate, how many servings of **walnuts** did you eat?

€ < 1 per week € 1-2 per week € 3 per week € 4 per week € 5-6 per week € 1 or more per day

4. How many servings of **fish or seafood** did you eat? Don’t include battered or fried products.

€ < 1 per week € 1-2 per week € 3 per week € 4-6 per week € 1 per day € 2 or more per day

4a. Of the fish and seafood that you ate, how many servings of **fatty fish** did you eat? *(e.g. salmon, tuna,*

*trout, sardines, herring, mackerel, anchovies)*

€ < 1 per week € 1 per week € 2-3 per week € 4-6 per week € 1 per day € 2 or more per day

1. How many servings of **canned beans or cooked dried beans** did you eat? *(e.g. chickpeas, lentils, kidney beans)*

 < 1 per week  1 per week  2-3 per week  4-6 per week  1 per day  2 or more per day

1. Did you eat **whole grain products** instead of white breads or refined grains? *(e.g. 100% whole grain breads, brown pasta, brown rice, bulgar, barley, oatmeal)*

 YES  NO  On most days

1. Did you eat **low-fat milk, yogurt, and cheese** instead of higher fat dairy products? *(e.g. 1% or skim milk, 0-2% yogurt, < 23% block cheese, feta cheese)*

 YES  NO  On most days

1. Did you use **extra virgin olive oil** as your main culinary oil? *(Used for cooking, in salad dressing, added to bread or other foods.)*

 YES  NO

8a. Over the past month, how many tablespoons of **extra virgin olive oil** did you usually consume

(including oil used in cooking, salad dressings, added to bread or any other food)?

€ Never € Less than once per month € 1-3 tbsp. per month € 1 tbsp. per week € 2-4 tbsp. per week

€ 5-6 tbsp. per week € 1 tbsp. per day € 2-3 tbsp. per day € 4-5 tbsp. per day € 6+ tbsp. per day

1. How many servings of **meat** **and poultry** (excluding fish) did you eat?

 < 1 per week  1-3 per week  4-6 per week  1 per day  2 per day  3 or more per day

9a. Of the meat that you ate, how many servings of **red or processed meat** did you eat? *(e.g. beef,*

*hamburger, pork, liver, lamb, veal, duck, ham, cold cuts, pepperoni sticks, jerky, pâté)*

€ < 1 per week € 1-3 per week € 4-6 per week € 1 per day € 2 per day € 3 or more per day

1. In total, how many servings of **butter, cream, hard-stick margarine, or high-fat dairy spreads** did you eat? *(Includes full-fat sour cream and cream cheese)*

 < 1 per week  1-3 per week  4-6 per week  1 per day  2 per day  3 or more per day

1. How many servings of **white bread** did you eat? *(e.g. white bread, rolls, bagels, pita, or tortilla)*

 < 1 per week  1 per week  2-3 per week  4-6 per week  1 per day  2 or more per day

12. Have you eaten any of the following foods over the past month?

€ YES € NO **Salty snacks and fried foods** (e.g. potato chips, pretzels, fries, egg rolls)

€ YES € NO **Sugary beverages** (e.g. pop, sweetened fruit juice, lemonade, sports drinks)

€ YES € NO **Candy, chocolates, bon bons**

€ YES € NO **Pre-packaged meals or sides** (e.g. frozen dinners or appetizers, canned soup)

€ YES € NO **Commercial** (not homemade) **baked goods**

€ YES € NO **Commercial** (not homemade) **dairy desserts**

12a. Think about the foods where you answered “YES”. In total, how many servings of these foods did

you usually eat?

€ < 1 per week € 1-2 per week € 3 per week € 4-6 per week € 1 per day € 2 or more per day

**Appendix 6: Exercise Session Feedback Form**

Your feedback on this research study is valuable and will help us to improve the next phase.

Please circle **one** answer for the following questions:

1. Did the exercise sessions provide you with new skills and/or useful knowledge?
   1. Yes b. No

Comments: __________________________________________________________________________________________________________________________________________

1. Were you provided with adequate information and resources to complete your exercise outside of the classes?
   1. Yes b. No

Comments:

____________________________________________________________________________________________________________________________________

1. Were you provided with adequate information and resources to continue your exercise routine after completion of the program?
   1. Yes b. No

Comments:

____________________________________________________________________________________________________________________________________

1. How did you find the length of the 1.5 hour weekly exercise sessions?
2. Just right b. Too long c. Too short

Comments:

____________________________________________________________________________________________________________________________________

1. Which part of the sessions did you find **most** interesting or useful?

________________________________________________________________________

________________________________________________________________________

________________________________________________________________________

________________________________________________________________________________________________________________________________________________________________________________________________________________________

1. Which part of the sessions did you find **least** interesting or useful?

________________________________________________________________________

________________________________________________________________________

________________________________________________________________________

________________________________________________________________________

________________________________________________________________________________________________________________________________________________

1. What were some of your challenges following the exercise program?

________________________________________________________________________________________________________________________________________________________________________________________________________________________________________________________________________________________________________________________________________________________________________________________________________________________________________________

1. Did you take advantage of any of the other services offered to you while participating in the program (e.g., Social worker, sleep/psychologist, diabetes lectures, etc.)
   - 1. Yes b. No

Describe:________________________________________________________________________________________________________________________________________________________________________________________________________________________________________________________________________________________________________________________________________________________________________________________________________________________________________

**Appendix 7: DIET session feedback form**

Your feedback on this research study is valuable and will help us to improve the next phase.

Please circle **one** answer for the following questions:

1. Did the nutrition sessions provide you with new skills and/or useful knowledge?
   1. Yes b. No

Comments: __________________________________________________________________________________________________________________________________________

1. Did the goal setting approach help you to make dietary changes?
   1. Yes b. No

Comments:

__________________________________________________________________________________________________________________________________________

1. Were you provided with adequate information and resources to follow the Brain Health Food Guide?
   1. Yes b. No

Comments:

____________________________________________________________________________________________________________________________________

1. How did you find the length of the one hour weekly nutrition sessions?
2. Just right b. Too long c. Too short

Comments:

____________________________________________________________________________________________________________________________________

1. Were the number of individual sessions provided with the dietitians:
   1. Adequate b. Too few c. Too many

Comments:

________________________________________________________________________ __________________________________________________________________

1. Which part of the sessions did you find **most** interesting or useful?

________________________________________________________________________

________________________________________________________________________

________________________________________________________________________

________________________________________________________________________________________________________________________________________________________________________________________________________________________

1. Which part of the sessions did you find **least** interesting or useful?

________________________________________________________________________

________________________________________________________________________

________________________________________________________________________

________________________________________________________________________

________________________________________________________________________________________________________________________________________________

1. What were some of your challenges following the Brain Health Food Guide?

________________________________________________________________________________________________________________________________________________________________________________________________________________________________________________________________________________________________________________________________________________________________________________________________________________________________________________

1. When this intervention is repeated, what should be left out, added or changed?

________________________________________________________________________________________________________________________________________________

________________________________________________________________________

________________________________________________________________________

________________________________________________________________________________________________________________________________________________

1. Would you recommend this research study to others concerned with their memory?
   - 1. Yes b. No

**Appendix 8: ED session feedback Form**

Your feedback on this research study is valuable and will help us to improve the next phase.

Please circle **one** answer for the following questions:

1. Did the group sessions provide you with new skills and/or useful knowledge?
   1. Yes b. No

Comments: __________________________________________________________________________________________________________________________________________

1. How did you find the length of the one hour weekly nutrition sessions?
2. Just right b. Too long c. Too short

Comments:

____________________________________________________________________________________________________________________________________

1. Were the number of individual sessions provided with the dietitians:
   1. Adequate b. Too few c. Too many

Comments:

________________________________________________________________________ __________________________________________________________________

Which part of the sessions did you find **most** interesting or useful?

________________________________________________________________________

________________________________________________________________________

________________________________________________________________________

________________________________________________________________________________________________________________________________________________________________________________________________________________________

1. Which part of the sessions did you find **least** interesting or useful?

________________________________________________________________________

________________________________________________________________________

________________________________________________________________________

________________________________________________________________________

________________________________________________________________________________________________________________________________________________

1. When this intervention is repeated, what should be left out, added or changed?

________________________________________________________________________________________________________________________________________________

________________________________________________________________________

________________________________________________________________________

________________________________________________________________________________________________________________________________________________

1. Would you recommend this research study to others concerned with their memory?
   - 1. Yes b. No

**Appendix 9: Full list of LEAD outcome assessments**

**Baseline visit 1 at Sunnybrook Hospital (~3 hours)**

- 6 Minute Walk Test (6MWT)
- Balance Assessment (on platform, eyes open, eyes closed)
- Gait Assessment (GAITRite: usual, fast, dual task)
- Autonomic battery (ECG, transcranial ultrasound, arterial tonometry, finger cuff blood pressure (while lying down, paced breathing, sit to stand, and squeezing handle))
- 5 times sit to stand
- Given Rumsey referral package

**Baseline visit 2 at Sunnybrook Hospital (~3 hours)**

Neuropsychological Assessment

- Direct Assessment of Functional Status - Revised (DAFS-R)
- Number-Letter task
- Beck Anxiety Inventory (BAI)
- Beck Depression Inventory II (BDI-II)
- Apathy’s inventory (participant)

Take home

- Toolbox test
- Geriatric Anxiety Inventory (GAI)

MRI

- 3D T1-weighted MRI
- PD/T2-weighted MRI
- FLAIR
- Gradient Echo
- Resting State fMRI (BOLD)
- DTI
- Attention-based task fMRI (BOLD) [LEAD specific functional task]

**Baseline visit 3 at the Cardiovascular Prevention and Rehabilitation Program (clinical program requirements) (~1.5 hours)**

- Resting ECG – screen for contraindications to exercise testing
- Review medical history and demographics
- Physical measures (height, weight, waist/hip circumference, Tanita body fat (BF) %, blood pressure (sitting, lying, orthostatic change))

**Baseline visit 4 at the Cardiovascular Prevention and Rehabilitation Program (~1 hour)**

- Graded exercise test

**Post-intervention/Follow-up visit 1 at Sunnybrook Hospital (~3 hours)**

- 6MWT
- Balance Assessment (on platform, eyes open, eyes closed)
- Gait Assessment (GAITRite: usual, fast, dual task)
- Grip strength
- 5 times sit to stand
- Autonomic battery (ECG, transcranial ultrasound, arterial tonometry, finger cuff blood pressure while lying down, paced breathing, sit to stand, and squeezing handle)

**Post-intervention/Follow-up visit 2 at Sunnybrook Hospital (~3 hours)**

Neuropsychological Assessment (* = pre-testing done as part of COMPASS-ND baseline) measures)

- Jessens Questions *
- MoCA *
- RAVLT – Imm. Recall *
- Reitan Trail Making test *
- Digit Symbol Substitution Test *
- RAVLT – Delay *
- Face-Name association – Imm. Recall *
- DKEFS Colour Word Interference *
- Face-Name association – delayed recall and recognition*
- DAFS-R
- Number-Letter task
- GDS*
- Apathy’s inventory (participant)
- Activities Specific Balance Confidence Scale *
- Sleep (Pittsburg Sleep Quality Index) *
- Cognitive Fluctuations (MAYO clinic fluctuations scale) *

Take home

- Toolbox test
- GAI
- QOL-AD scale

MRI

- 3D T1-weighted MRI
- PD/T2-weighted MRI
- FLAIR
- Gradient Echo
- Resting State fMRI (BOLD)
- DTI
- Attention-based task fMRI (BOLD) [LEAD specific functional task]

**Post-intervention/Follow-up visit 3 at the Cardiovascular Prevention and Rehabilitation Program (~1.5 hours)**

- Physical measures (height, weight, waist/hip circumference, BF %, blood pressure (sitting, lying, orthostatic change)
- Graded exercise test
